# Editorial: Advances in stem cell therapy: new applications and innovative therapeutic approaches

**DOI:** 10.3389/fmed.2023.1225551

**Published:** 2023-08-08

**Authors:** Annalisa Marcuzzi, Natalia Maximova

**Affiliations:** ^1^Department of Translational Medicine, University of Ferrara, Ferrara, Italy; ^2^Department of Pediatrics, Bone Marrow Transplant Unit, Institute for Maternal and Child Health-IRCCS Burlo Garofolo, Trieste, Italy

**Keywords:** allogeneic cell cancer immunotherapy, induced pluripotent stem cells (iPSCs)-derived cell therapy, gene-engineered T cells, virus-specific cytotoxic T lymphocytes, extracellular vesicle-based therapies, disease-modifying therapy, autoimmune diseases

Stem cell therapy has continued to advance, bringing hope to cure diseases that were once considered incurable. The concepts underlying the use of stem cells in therapy depend on their inherent capacity for regenerating the original tissues of the body. Additionally, stem cells can be altered to provide powerful drugs or nanomaterials and have an ability to modulate the immune system. Moreover, innovative advances continue in immunotherapy with allogeneic cells and their progress toward clinical use (1). The T cell immunology field has focused on cytotoxic T lymphocytes, which play an essential role in the immune defense against viral infections and malignancies (2). Few stem cell therapies are currently approved and have been integrated into standard clinical protocols.

The most widely adopted stem cell therapy is the transplantation of hematopoietic stem cells to treat hematological malignancies and disorders of the immune system and blood. Other ongoing clinical trials involving stem cell therapies have already generated impressive results, such as when patient-derived induced pluripotent stem cells (iPSCs) were induced to differentiate into pigment epithelial cells of the retina when transplanted into patients with macular degeneration, greatly improving the patient's sight (3). Furthermore, in a recent world-first, iPSC-derived mesenchymal stromal cells have successfully been used to treat patients with acute steroid-resistant graft vs. host disease (4).

One of the newest and most promising immunotherapy-based treatments against solid tumors and hematological malignancies are chimeric antigen receptor (CAR)-based therapies. They have produced remarkable clinical responses in patients with B-cell leukemia or lymphoma. However, there are important limitations to CAR-T cell therapy, including toxicities related to life-threatening adverse events, limited effectiveness against solid tumors, inhibition and resistance in B-cell malignancies, and the tumor antigen escape (5). Furthermore, the *ex-vivo* production of CAR-T cells is a very complex, long, and costly process. As discussed in the first article, Wakao and Fukaya-Shiba, members of the Center for Regulatory Science, Pharmaceuticals and Medical Devices Agency of Japan, presented an opinion article describing the significant obstacles and solutions currently proposed to overcome and optimize CAR-T therapy. To overcome the difficulties of current *ex vivo* CAR-T cell therapy approaches, intensive research efforts are in place to generate CAR-T cells *in vivo* through direct immune-gene vector injection to make this treatment immediately accessible to patients (Wakao and Fukaya-Shiba). The purpose of this article is to inform general readers about the regulatory challenges in the development of *in vivo*-generated CAR-T products.

Another interesting CAR-T cell-based approach that allows one to get around the hurdles of CAR-T cell therapy, such as their limited persistence, poor trafficking, and tumor infiltration, is discussed in the second review article of Osorio-Rodríguez et al.. Immunotherapy with autologous T-cells engineered to express the receptor tyrosine kinase-like orphan receptor 1-specific chimeric antigen receptor (ROR1) CAR-T cells has described a therapeutic option for patients with tumor recurrence after conventional treatments because some hematological malignancies and solid tumors overexpress ROR1.

Neurological disorders are recognized as the leading causes of death and disability worldwide and represent one of the most significant public health challenges. There is renewed research effort to identify new, more effective treatment methods for neurological patients (6). One of these methods is based on stem cell therapies. Notably, mesenchymal stem cell (MSC) therapy has appeared as a promising strategy due to its excellent properties, such as simple isolation, multipotent differentiation potential, and powerful paracrine activity. In the third original research, Ercelen et al. describe their experience treating stroke patients using allogeneic umbilical cord MSCs. Significant improvements in clinical outcomes have been observed in the general clinical conditions of patients treated with the umbilical cord MSCs. In addition, the authors reported an improvement in muscle strength, spasticity, and fine motor functions documented in all treated patients. This study is important in demonstrating neural protection and recovery through the anti-inflammatory and immunomodulation effects of MSCs for treating stroke in acute and chronic periods. Another example of stem cell use for regenerative purposes is reported in the case report by Mao et al. in the treatment of femoral head osteonecrosis. The authors describe the migration of peripheral blood stem cells (PBSCs), labeled with 2-[18F]-fluoro-2-deoxy-D-glucose (18F-FDG tracer) and infused through the medial circumflex femoral artery (7). 3D-PET imaging showed that although PBSCs labeled with 18F-FDG were widely distributed around the hip, such as the femoral bone marrow cavity, femoral head, and acetabulum, PBSCs were generally located in the necrotic area of the femoral head.

As innovative bioengineering technologies have matured sufficiently for the commercialization phase, there has been considerable investment in cell development and gene therapy in recent years, resulting in growing numbers of clinical studies in the field (6). In addition, a string of regulatory and legislative tasks has recently been introduced to regulate the latest pharmaceutical products systematically. Accordingly, another level of legislation and tailor-made policies for cell and gene therapies has been introduced, which should evolve along with technological progress (8). To stay current with regulations, the manual search process must be repeated periodically, reviewing the previous evaluation to ensure that updated rules are retrieved and brought to the repository. Schaut et al. present an automated search system for exploration of regulations related to the production of cell and gene therapy products. The objective of developing this custom automated retrieval system was to increase delivery of recently published applicable regulations and improve the quality of results. This would instill confidence that appropriate regulations would be identified and outputs obtained rapidly and more economically than the current manual process.

In recent years, the use of stem cells has expanded into multiple fields of medicine. Experiments have been performed to generate insulin-secreting cells, neural cells, heart cells, and other tissue-specific cells *ex vivo* and in experimental animals. Subsequently, many clinical trials followed these exploratory tests in specific pathologies. In the not-too-distant future, stem cell therapies will make it possible to treat numerous pathologies that are incurable today successfully.

## Author contributions

NM wrote the editorial. AM critically revised the editorial. All authors contributed to the article and approved the submitted version.
